# Bringing Ontology to the Gene Ontology

**DOI:** 10.1002/cfg.253

**Published:** 2003-02

**Authors:** Jennifer Williams, William Andersen

**Affiliations:** 1 Ontology Works Inc. 1132 Annapolis Road Suite 104 Odenton MD 21113 USA

## Abstract

We present an analysis of some considerations involved in expressing the Gene Ontology (GO) as a machine-processible ontology, reflecting principles of formal ontology. GO is a controlled vocabulary that is intended to facilitate communication between biologists by standardizing usage of terms in database annotations. Making such controlled vocabularies maximally useful in support of bioinformatics applications requires explicating in machine-processible form the implicit background information that enables human users to interpret the meaning of the vocabulary terms. In the case of GO, this process would involve rendering the meanings of GO into a formal (logical) language with the help of domain experts, and adding additional information required to support the chosen formalization. A controlled vocabulary augmented in these ways is commonly called an *ontology*. In this paper, we make a modest exploration to determine the ontological requirements for this extended version of GO. Using the terms within the three GO hierarchies (molecular function, biological process and cellular component), we investigate the facility with which GO concepts can be ontologized, using available tools from the philosophical and ontological engineering literature.


**Comparative and Functional Genomics**
*Comp Funct Genom* 2003; **4**: 90–93.
Published online in Wiley InterScience (www.interscience.wiley.com). **DOI:** 10.1002/cfg.253

**Conference Review**

### Bringing Ontology to the Gene Ontology

Jennifer Williams* and William Andersen
*Ontology Works Inc., 1132 Annapolis Road, Suite 104, Odenton, MD 21113, USA*

**Correspondence to:
Jennifer Williams, Ontology Works Inc., 1132 Annapolis Road, Suite 104, Odenton, MD 21113, USA.
E-mail: williams@ontologyworks.com*

Received: 30 October 2002
Accepted: 3 December 2002

### Abstract

**We present an analysis of some considerations involved in expressing the Gene Ontology (GO) as a machine-processible ontology, reflecting principles of formal ontology. GO is a controlled vocabulary that is intended to facilitate communication between biologists by standardizing usage of terms in database annotations. Making such controlled vocabularies maximally useful in support of bioinformatics applications requires explicating in machine-processible form the implicit background information that enables human users to interpret the meaning of the vocabulary terms. In the case of GO, this process would involve rendering the meanings of GO into a formal (logical) language with the help of domain experts, and adding additional information required to support the chosen formalization. A controlled vocabulary augmented in these ways is commonly called an *ontology*. In this paper, we make a modest exploration to determine the ontological requirements for this extended version of GO. Using the terms within the three GO hierarchies (molecular function, biological process and cellular component), we investigate the facility with which GO concepts can be ontologized, using available tools from the philosophical and ontological engineering literature. Copyright © 2003 John Wiley & Sons, Ltd.**

### Introduction

The rapidly increasing wealth of genomic data has driven the development of computer systems to assist in the biologist's task of correlating related knowledge about genes, their products and their functions [8]. Gene products must be annotated according to generally accepted and coherent theories of biology.

We feel that there are benefits to expressing these theories as ontologies that are both (a) formalized in machine-processible language and (b) reflect principles of formal ontology (e.g. [11]). The utility of ontologies in bioinformatics is well accepted [12], and formalization of ontologies allows the use of computers to verify internal consistency and consistency with available data [6]. Indeed, formalization of GO is being pursued by the GONG project [3]. The utility of applying formal ontology to bioinformatics problems is more speculative, in that the relevant principles have been introduced from philosophy relatively recently (e.g. [5]), but we feel that formal ontology suggests essential *domain-independent* organizing principles.

We explore examples of applying some of these principles to GO, with a view toward improving GO's consistency and general applicability to automated processing of biological data. After a brief introduction to GO, we seek to classify GO terms according to groups of mutually disjoint ontological categories, such as *universal* (classes and relationships) vs. *individual* object, or *continuant* (everyday objects) vs. *occurrent* (events or processes) and their organization by higher-order properties (e.g. 'species'). We attempt to uncover the semantics of the GO relations *is-a* and *part-of.* We conclude with future directions for this work.

### The Gene Ontology (GO)

GO [1] is a source of generally accepted biological theory of considerable breadth. GO, the product

Copyright © 2003 John Wiley & Sons, Ltd.

of a consortium of researchers working on several model organism databases, has the stated goal, 'to provide a set of structured vocabularies for specific biological domains that can be used to describe gene products in any organism', to 'facilitate communication between people and organizations' [2]. GO's scope is larger and its structure more complex than many other classification schemes [9]; thus, it provides a rich resource for a project seeking to ontologize biological theory.

The GO Consortium states, 'Curatorially, it will be increasingly difficult to maintain the semantic consistency we desire without software tools that perform consistency checks and controlled updates', and notes that 'Many molecular functions and biological processes do not exist in all organisms' [2]. Both of these concerns can be addressed to some extent by an ontology that can apply different *rules* (statements about the way the world works) to different species, and which checks for semantic consistency between all of the rules expressed in the ontology.

### Discussion

We seek to ontologize the semantics of GO, as implied by its documentation and content. The GO consists of three sets of terms (cellular component, molecular function and biological process), arranged in directed acyclic graphs (DAGs), connected by 'is-a' and 'part-of' relations. (DAGs are distinct from hierarchies, in that each term in a DAG may have more than one parent term; however, we use the colloquial 'hierarchy' equivalently to 'DAG' in this work.)

### Basic ontological distinctions

#### Universal vs. individual

GO terms represent abstractions. The cellular component ontology, for example, models the general structure of eukaryotic cells [1], rather than any actual individual cells. Such terms are typically taken to name *properties* — a kind of *universal* that accounts for the common structure of objects. Properties may be *exemplified* or *instantiated* by objects, while *individuals* may not [7]. Since each GO term may be instantiated by some real-world object, e.g. the term *Cell* could be instantiated by some actual cell in a Petri dish, we know that GO terms cannot be individuals.

#### Continuant vs. occurrent

The cellular component hierarchy contains terms representing kinds of physical objects (e.g. 'dendrite'), whereas the biological process and molecular function hierarchies contain terms representing events (e.g. 'budding'). Physical objects (*continuants*) should be distinguished from events (*occurrents*) in order to account for their different ways of behaving, e.g. events can only have physical objects as *participants* in the event, not as *parts* of the event (which could only be sub-events). For example, 'ubiquitin ligase complex' (a cellular component) would be represented as a *participant* in 'protein ubiquitylation' (a biological process), not as a *part of* it.

### GO relations

#### The semantics of 'is-a'

The relation of *subsumption* captures the notion that one property is more general than another (e.g. 'cell *subsumes* eukaryotic cell'), whereas instantiation reflects that an entity exemplifies a property ('my cat is an *instance of* cat'). We note that 'is-a' relates only GO terms (already determined to be properties), and that its behaviour is transitive (e.g. if lipoprotein antitoxin is a kind of antitoxin, and antitoxin is a kind of molecular function, then lipoprotein antitoxin is a kind of molecular function). Therefore, we take the intended meaning to be *subsumption between properties*, rather than instantiation of properties.

#### The semantics of 'part-of'

The intended meaning of 'part-of' can be found in the GO Usage Guide [4]: part-of indicates '*can be a part of*', NOT '*is always a part of*'. The most natural reading of 'part-of' is that 'Property $A$ is part-of Property $B$ only when, for instances $a$ of $A$ and $b$ of $B$, it is *possible* that $a$ is part of $b$'. This is distinct from a subsumption relationship, in which, if Property $A$ is subsumed by Property $B$, all instances of $A$ are *necessarily* instances of $B$. The behaviour of 'part-of' is defined as transitive. Perusing GO shows examples of 'part-of' used for representation of, at least, parts (steps) of processes, parts of functions, and physical parts, as well as in each of the following kinds of conceptualizations:

- 'A membrane is a part of (**any**) cell'.
- 'A flagellum is part of (**some**) cells'.

Copyright © 2003 John Wiley & Sons, Ltd.

- 'A replication fork is part of the nucleoplasm (**only** during certain times of the cell cycle)'.

Each variant of this variety of usages should ideally be represented as a different 'flavour' of 'part-of'; however, until these semantics can be formalized, a very general representation of 'part-of' is needed.

#### Non-rooted terms

Examination of the GO ontologies (July 2002 release) shows that about 1200 terms lack an 'is-a' parent, e.g. 'inner membrane' is part-of 'membrane' but is not subsumed by anything. Therefore, it lacks a 'definition', in the sense that 'the definition of a concept becomes the [subsumption] path from its own node to the root node of the ontology' [10]. Because each term is semantically rooted in its GO hierarchy, we can automatically subsume terms with 'cellular component', 'molecular function' or 'biological process' (e.g. 'membrane' would be subsumed by 'cellular component'). This 'rooting' of properties is not a requirement of formal ontology; however, we find that the process of ontologization encourages more complete definition of properties.

### Higher-order properties

#### Implied higher-order properties

Just as individual objects ('my cat Fluffy') may be grouped under a property ('Cat'), properties may likewise be grouped under properties. These latter properties are called higher-order properties, and in general are called higher-order relations (e.g. subsumption) if they relate properties or relations. Gathering collections of properties into higher-order properties allows the modelling of such concepts as *species*:

- 'Spot' is an instance of *Canis familiaris.*
- *C. familiaris* is an instance of the higher-order property Species.
- (But 'Spot' is not an instance of Species!).

Higher-order properties are implied by the structure of GO, e.g. every term in the cellular component hierarchy represents, naturally, a *kind of cellular component*, such as 'membrane'. We can therefore identify each cellular component term as an *instance of* cellular-component-kind (similarly for molecular-function-kind and biological-process-kind). This facilitates modelling tasks such as indicating that plant cells and animal cells have different *kinds* of cellular components.

Higher-order properties enable us to specify certain relations that are relevant for certain kinds of entities, such as modelling the *kinds of processes* that are restricted to certain *kinds of cellular components*. For example, we generalize our observation that each individual photosynthesis reaction takes place in the thylakoid compartment of an individual cell; it is in fact the case that *the kind of process* called photosynthesis always takes place in the *kind of cellular component* called thylakoid.

#### Types

There is another formal aspect of molecular function, cellular component and biological process terms. An instance of a cellular component, e.g. a particular nucleus, is expected to remain a nucleus and to be distinguishable from other nuclei. The ontology in [5] identifies such non-varying (in formal ontological terms, 'rigid') and distinguishable ('identity-carrying') entities as instances of the higher-order property *Type*. We believe that molecular function and biological process properties are also distinguishable and rigid, therefore molecular function and biological process properties will also be instances of *Type*. Ideally, every real-world object will instantiate some Type, which serves as a source of distinguishing identity characteristics for the object.

### Proposed future directions

There are a few difficulties with GO that could be remedied by knowledge-intensive reformulation and/or extension. For example, the GO Usage Guide [4] states that the following must be true:

> If A is part of B, and C is an instance of B, is A part of C? — YES.

Examining the Gene Ontology, one sees such cases as:

> Axon is part-of Cell, and Ascus is an 'instance of' (i.e. subsumed by) Cell, therefore Axon is part-of Ascus.

Since an Ascus is a fungal cell, it is not possible that Axon (part of a neuron) can even *potentially* be part of it. This problem also exists for Glycosome (part of Kinetoplastida cells), Viral Tegument (part of viral particles), etc.

Copyright © 2003 John Wiley & Sons, Ltd. 

Possible solutions include extending the cellular ontology to include the needed specific kinds of cells: e.g. Neuron, of which Axon may be a part; and Kinetoplastida-cell, of which Glycosome may be a part. Creating a kind-of-cell (e.g. neuron, muscle cell) ontology would be helpful; but rather than proliferating such properties as Kinetoplastida-cell, Mammalian-cell, Chordate-cell, it would make more sense to additionally create a zoological ontology which would be referred to in statements such as, 'glycosome can be part of a cell *for Kinetoplastidae*'. The utility of this extension is evident; variability between organisms is so great that it is necessary to restrict statements to specific organisms, e.g. 'notochord is found in some developmental stage *in chordates*.'

Further ontologization based on GO would likely include such extensions as:

- Formalize the different semantics of the 'annotation' relation between gene products and kinds of GO terms; e.g. the relation between a gene product and a cellular component might be 'active-in-component' [2].
- Enrich GO's ontological content to extend its capabilities, e.g. an ontology of biological substances could be used to infer that 'macromolecule' subsumes 'polysaccharide', which subsumes 'chitin'. Therefore, 'chitin catabolism' could be automatically inferred to be a kind of 'macromolecule metabolism', rather than the curator making all of these connections manually. As more biological concepts are represented formally, computer systems can begin to assume more of the burden of ontology curation by enforcing consistency in the semantics of the ontology, as expressed in concept hierarchies and in rules.
- The GO Usage Guide [4] states: 'A biological process is a biological goal that requires more than one function'. This, and other, relations between the GO ontologies should be developed, e.g. the relationship between 'apoptosis inhibitor' and 'apoptosis inhibition', or between 'flagellum' (cellular component) and 'cellular motility' (biological process). Knowledge about where processes may take place should be recorded, e.g. photosynthetic reactions take place *in thylakoid*, as well as temporal associations, e.g. nucleolus reappearance is associated with *telophase* of mitosis. These between-hierarchy relations are characteristic of ontologies and would allow the capture of more biological knowledge.

This work consisted of an initial analysis of GO terms and organization with respect to some common formal ontological notions. Future work would include the actual formalization of some part of GO in a logical language (and the potential use of software to verify the internal semantic consistency of the ontology), adding some extensions to GO as suggested by the ontological analysis, and examining GO with respect to other aspects of formal ontological theory (e.g. mereology, constitution, granularity). Future analysis and extensions will preferably be performed in consultation with experienced GO users and GO experts.

In conclusion, projects directed toward extending the computational capabilities of bioinformatics systems have a tremendously valuable starting point in the GO. Guidance provided by the discipline of formal ontology can help such projects make principled initial ontologization decisions that support the intended semantics of GO.

### References

1. Gene Ontology Consortium. 2000. Gene ontology: tool for the unification of biology. *Nature Genet* **25**: 25–29.
2. Gene Ontology Consortium. 2001. Creating the gene ontology resource: design and implementation. *Genome Res* **11**: 1425–1433.
3. GONG Project: **http://gong.man.ac.uk**
4. GO Usage Guide: **http://www.geneontology.org/GO.usage.html**
5. Guarino N, Welty C. 2000. Ontological analysis of taxonomic relationships. In *Proceedings of ER-2000*. Laender A, Story V (eds). Springer-Verlag LNCS: New York.
6. Karp PD. 2001. Pathway databases: a case study in computational symbolic theories. *Science* **293**: 2040–2044.
7. Loux MJ. 1998. *Metaphysics: a Contemporary Introduction*. Routledge: New York.
8. Reed J. 2000. Trends in commercial bioinformatics. *Oscar Gruss Biotechnol Rev* **13**(March).
9. Rison SCG, Hodgman TC, Thornton JM. 2000. Comparison of functional annotation schemes for genomes. *Funct Integr Genomics* **1**: 56–69.
10. Schulze-Kremer S. 1998. Ontologies for molecular biology. In *Pacific Symposium on Biocomputing*. AAAI Press: 695–706.
11. Simons P. 1987. *Parts: a Study in Ontology*. Oxford University Press: Oxford.
12. Stevens R, Goble C, Bechhofer S. 2000. Ontology-based knowledge representation for bioinformatics. *Brief Bioinform* **1**(4): 398–414.

Copyright © 2003 John Wiley & Sons, Ltd.
